# Improvement of Z-Weighted Function Based on Fifth-Order Nonlinear Multi-Order Weighted Method for Shock Capturing of Hyperbolic Conservation Laws

**DOI:** 10.3390/e26040334

**Published:** 2024-04-14

**Authors:** Jinwei Bai, Zhenguo Yan, Meiliang Mao, Yankai Ma, Dingwu Jiang

**Affiliations:** 1 Computational Aerodynamics Institute, China Aerodynamics Research and Development Center, Mianyang 621000, China; 2 State Key Laboratory of Aerodynamics, China Aerodynamics Research and Development Center, Mianyang 621000, China; yanzhg@mail.ustc.edu.cn (Z.Y.);

**Keywords:** nonlinear weighted scheme, multi-order weighted method, shock capturing, WCNS (weighted compact nonlinear scheme), gas dynamics

## Abstract

Based on a 5-point stencil and three 3-point stencils, a nonlinear multi-order weighted method adaptive to 5-3-3-3 stencils for shock capturing is presented in this paper. The form of the weighting function is the same as JS (Jiang–Shu) weighting; however, the smoothness indicator of the 5-point stencil adopts a special design with a higher-order leading term similar to the τ in Z weighting. The design maintains that the nonlinear weights satisfy sufficient conditions for the scheme to avoid degradation even near extreme points. By adjusting the linear weights to a specific value and using the τ in Z weighting, the method can be degraded to Z weighting. Analysis of linear weights shows that they do not affect the accuracy in the smooth region, and they can also adjust the resolution and discontinuity-capturing capability. Numerical tests of different hyperbolic conservation laws are conducted to test the performance of the newly designed nonlinear weights based on the weighted compact nonlinear scheme. The numerical results show that there are no obvious oscillations near the discontinuity, and the resolution of both the discontinuity and smooth regions is better than that of Z weights.

## 1. Introduction

Low-order schemes have been widely adopted to solve hyperbolic equations to avoid non-physical oscillations near discontinuities [1]. However, the relatively large dispersion and dissipation errors of low-order schemes will smear out a lot of flow details in smooth regions [2], which has promoted the development of high-order nonlinear schemes. Representative achievements of high-order nonlinear schemes include ENO (essentially non-oscillatory) [3], WENO (weighted ENO) [4], TENO (targeted ENO) [5], CNS (compact nonlinear scheme) [6], WCNS (weighted CNS) [7], TCNS (targeted CNS) [8], etc. Nonlinear weighting has played an important role in developing some of these schemes, which provide largely improved scheme accuracy and computational efficiency.

The research on nonlinear weighted schemes has lasted for nearly 30 years. Many kinds of nonlinear weighting functions based on the WENO scheme have been proposed for better accuracy in the smooth region and better non-oscillatory properties near discontinuities. They mainly focus on improving the three main components of a nonlinear weighting scheme, which are the candidate interpolation choice, smoothness indicator, and nonlinear weighting function. Research focusing on improving candidate interpolation choices includes the bandwidth optimization of Martin et al. [9] and the adaptive central-upwind weighted scheme of Hu et al. [10]. Meanwhile, Ha [11] modified the calculation of smoothness indicators, the special contribution of which lies in the parameter that adjusts the contribution of the first-order difference and the second-order difference to the smoothness indicators. Henrick [12] improved the relationship between the smoothness indicator and the nonlinear weight function by introducing an extra mapping function. Borges [13] reconstructed the smoothness indicators of each sub-stencil by introducing a higher-order smoothness indicator, thereby altering the expression form of the weight function. This approach has subsequently been extensively studied and applied by numerous researchers. The review papers [14,15,16] give a relatively comprehensive overview of the development of the WENO schemes.

Another branch of nonlinear weighted schemes is the weighted compact nonlinear scheme (WCNS) [7,17,18,19], which is the scheme the current research is based on. It was summarized to have a series of advantages over the WENO schemes [2]. A key property that distinguishes WCNS from WENO is that it has some advantages in the simulation of complex geometries [20] because of satisfying the geometric conservation law [21,22,23,24]. Note that WCNS and WENO share similar nonlinear weighting processes; therefore, nonlinear weights for one of them can be easily generalized to another.

The improvements in the weighting functions share a common goal, which is to maintain accuracy in smooth regions and capture discontinuities without introducing obvious oscillations. After the proposal of the original fifth-order WENO-JS [25] scheme, an issue of order reduction near extreme points was observed. As a result, several researchers have made a series of modifications [9,10,11,12,13,26] to further improve the performance of the weighting functions. Among these improved weighting functions, Zhu et al. [27] proposed the WENO-ZQ scheme by adopting sub-stencils of different sizes, which is different from the WENO-JS scheme using same-size sub-stencils. The sub-stencils of WENO-ZQ consist of one 5-point sub-stencil along with two upwind and central 2-point sub-stencils, which is represented by the notation (5-2-2) for simplicity. The WENO-ZQ scheme can achieve optimal order even at extreme points, but it still exhibits non-physical oscillations near strong discontinuities. Subsequently, the WENO-MR (5-3-1) scheme [28,29] was proposed based on the combination of (5-3-1) sub-stencils, which reduced non-physical oscillations near discontinuities and enhanced robustness. However, it is important to note that the introduction of the first-order sub-stencil may potentially result in decreased resolution. Zhang [30] applied a similar strategy of [28] to WCNS, resulting in the WCNS-MR (5-3-1) scheme, which exhibits better resolution in smooth regions compared to the WENO-MR (5-3-1) scheme. However, there are still significant non-physical oscillations near strong discontinuities. Wang [31] further improved the efficiency of the WCNS-MR scheme. In the above research, except for the 5-point sub-stencil, the interpolation accuracy of the remaining sub-stencils is very low. To ensure accuracy, not only will the design complexity of the nonlinear weight function increase, but the linear weight of the 5-point stencil in actual examples also needs to be close to 1. This linear weight deteriorates the ability to capture discontinuities. Since its proposal, the Z function has been widely used and provides good stability and resolution near discontinuities. However, as the complexity of the problem increases, it is necessary to obtain more flow details in smooth regions, and the resolution of Z weights can no longer meet practical needs.

The goal of this article is to propose a new nonlinear weighted format based on the WCNS-JS and 5-3-3-3 stencils. Its characteristic feature is that the order of smoothness indicator on the stencil is related to the highest interpolation accuracy of the stencil, which maintains that nonlinear weights meet sufficient conditions to ensure that the scheme does not degrade in accuracy near extreme points. The smoothness indicator of the 5-point sub-stencil is obtained by combining the smoothness indicators of two 3-point sub-stencils and with an order higher than that of the 3-point sub-stencil. In addition, the ratio of linear weights on the three 3-point stencils meets the requirements of convex combination to fifth-order accuracy. A theoretical derivation and numerical tests are conducted to compare the resolution of the weighted method with Z nonlinear weights. These tests demonstrate the new weighted method’s ability to achieve the desired accuracy in the smooth region and near the first- and second-order extreme points as well as its capability to accurately capture discontinuities.

The organization of the paper is as follows: In Section 2, we briefly introduce the fifth-order WCNS scheme. In Section 3, we establish a multi-order nonlinear weighted interpolation method based on the 5-3-3-3 stencils. In Section 4, some traditional numerical examples are used to demonstrate the characteristics of the multi-order weighting strategy in terms of smooth region resolution and discontinuous capture. In Section 5, we provide the conclusions obtained in this article.

## 2. WCNS

We focus on hyperbolic conservation laws of the form

(1) $$\frac{\partial u}{\partial t}+\frac{\partial f(u)}{\partial x}=0.$$

The WCNS procedure consists of three components: a high-order flux difference scheme, numerical flux evaluation, and weighted nonlinear interpolation. WCNS-E5 [32] is adopted for spatial discretizations, in which explicit flux differencing is used to calculate the ∂f/∂x term in Equation (1), which is

(2) $$f_{j}^{\prime }=\frac{1}{h}\left[a_{0}\left({\hat{f}}_{j+1/2}-{\hat{f}}_{j-1/2}\right)+a_{1}\left({\hat{f}}_{j+3/2}-{\hat{f}}_{j-3/2}\right)+a_{2}\left({\hat{f}}_{j+5/2}-{\hat{f}}_{j-5/2}\right)\right],$$

where $a_{0}=75/64$, $a_{1}=-25/384$, $a_{2}=3/640$, ${\hat{f}}_{j+l+1/2}$ is the numerical flux obtained at cell-edge, and $f_{j}^{\prime }$ represents a numerical approximation to the spatial derivative of the numerical flux *f* at the *j* cell-node. Equation (2) is sixth-order in accuracy with the parameters adopted.

In the WCNS, the flux at the half nodes is calculated using the flow variables ${\hat{u}}_{j+l+1/2}^{+}$ and ${\hat{u}}_{j+l+1/2}^{-}$, which are interpolated based on variables on the cell nodes. Numerical dissipation and the capture of discontinuities can be achieved through the use of either linear or nonlinear upwind interpolation and a suitable choice of the numerical flux. The interpolations of ${\hat{u}}_{j+l+1/2}^{+}$ and ${\hat{u}}_{j+l+1/2}^{-}$ are symmetric to each other. For clarity, the superscripts of + and − are dropped, and only the formulas for ${\hat{u}}_{j+l+1/2}^{+}$ are presented.

To ensure fifth-order accuracy of the overall scheme in smooth regions, the interpolation of ${\hat{u}}_{j+l+1/2}$ also requires fifth-order accuracy. The WCNS-E5 scheme designs a nonlinear interpolation method with an ideal accuracy of fifth-order on the stencil $\left(x_{j+l-2},x_{j+l-1},x_{j+l},x_{j+l+1},x_{j+l+2}\right)$. The specific implementation of the interpolation process is shown in Section 3.

## 3. Nonlinear Multi-Order Weighted Interpolation

The nonlinear weighted interpolation procedure in WCNS is very similar to the weighted reconstruction in popular schemes such as WENO, TENO, etc. These weighted nonlinear procedures commonly incorporate smoothness indicators on sub-stencils to effectively detect and capture discontinuities. In theory, the research results obtained can be generalized to other nonlinear weighting schemes as well.

After first introducing the general nonlinear weighted interpolation procedure, the classical JS nonlinear interpolation and the Z nonlinear interpolation will be presented in Section 3.1 and Section 3.2, respectively. After that, the newly proposed weighting method is presented in Section 3.3, followed by its spectral analysis in Section 3.4.

The Lagrangian interpolation expression for WCNS-E5 based on 5-point stencil

$S=\left\{x_{j-2},x_{j-1},x_{j},x_{j+1},x_{j+2}\right\}$ is

(3) $$u_{j+1/2}^{linear}=\frac{1}{128}\left(3u_{j-2}-20u_{j-1}+90u_{j}+60u_{j+1}-5u_{j+2}\right).$$

The above interpolation is decomposed into a combination of three third-order interpolations based on 3-point sub-stencils $S_{1}=\left\{x_{j-2},x_{j-1},x_{j}\right\}$, $S_{2}=\left\{x_{j-1},x_{j},x_{j+1}\right\}$, and $S_{3}=\left\{x_{j},x_{j+1},x_{j+2}\right\}$. The specific form of the combination and the third-order interpolations are

(4) $$\begin{array}{cc} \; & u_{j+1/2}^{linear}=d_{1}u_{j+1/2}^{1}+d_{2}u_{j+1/2}^{2}+d_{3}u_{j+1/2}^{3}=u_{j+1/2}+O\left(h^{5}\right),\\ & u_{j+1/2}^{1}=\frac{1}{8}\left(3u_{j-2}-10u_{j-1}+15u_{j}\right)=u_{j+1/2}+O\left(h^{3}\right),\\ & u_{j+1/2}^{2}=\frac{1}{8}\left(-u_{j-1}+6u_{j}+3u_{j+1}\right)=u_{j+1/2}+O\left(h^{3}\right),\\ & u_{j+1/2}^{3}=\frac{1}{8}\left(3u_{j}+6u_{j+1}-u_{j+2}\right)=u_{j+1/2}+O\left(h^{3}\right), \end{array}$$

where the ideal weights are $d_{1}=1/16$, $d_{2}=10/16$ and $d_{3}=15/16$, resulting in a convex combination with fifth-order accuracy. The functions $u_{j+1/2}^{1}$,$u_{j+1/2}^{2}$, and $u_{j+1/2}^{3}$ are third-order interpolations for the $S_{1}$, $S_{2}$,and $S_{3}$ stencils, respectively. Similar to the linear weighted convex combination expression in Equation (4), nonlinear weighted interpolation is written as

(5) $$u_{j+1/2}^{nonlinear}=\omega_{1}u_{j+1/2}^{1}+\omega_{2}u_{j+1/2}^{2}+\omega_{3}u_{j+1/2}^{3}$$

### 3.1. JS Nonlinear Interpolation

The famous JS nonlinear weights from Jiang and Shu [25] are defined as

(6) $$\omega_{k}=\frac{\alpha_{k}^{JS}}{\sum_{i=1}^{3}\alpha_{i}^{JS}},\;\alpha_{k}^{JS}=\frac{d_{k}}{{\left(\beta_{k}^{JS}+\varepsilon \right)}^{q}}$$

where the constant ε in the above equation is a small quantity to avoid the denominator becoming 0, and $\beta_{k}$ is the smoothness indicator. Usually, *q* takes a value of 2. The term $\beta_{k}^{JS}$ [14] is defined as

(7) $$\left\{\begin{array}{c} \beta_{1}^{JS}=\frac{1}{4}{\left(u_{j-2}-4u_{j-1}+3u_{j}\right)}^{2}+{\left(u_{j-2}-2u_{j-1}+u_{j}\right)}^{2}\\ \beta_{2}^{JS}=\frac{1}{4}{\left(u_{j+1}-u_{j-1}\right)}^{2}+{\left(u_{j-1}-2u_{j}+u_{j+1}\right)}^{2}\\ \beta_{3}^{JS}=\frac{1}{4}{\left(-3u_{j}+4u_{j+1}-u_{j+2}\right)}^{2}+{\left(u_{j}-2u_{j+1}+u_{j+2}\right)}^{2} \end{array}\right.$$

Previous studies have shown that such nonlinear weights can lead to a decrease in interpolation accuracy at the extreme points in smooth regions, resulting in a decrease in the overall scheme accuracy. To address this issue, numerous scholars have conducted extensive research, proposing the mapped WENO (M) [12], Z nonlinear weights [13], and ZQ nonlinear weights [27], etc.

### 3.2. Z Nonlinear Interpolation

Z nonlinear weights proposes [13] a high-order smoothing indicator and uses it to provide a construction method of smoothing indicators that differs from JS, theoretically solving the problem of extreme point reduction. Although Z weights are proposed based on the WENO format, this idea can be ported to nonlinear interpolation methods in the WCNS scheme. A brief introduction is as follows:

The calculation method for the smoothness indicator of each stencil is Equation (7), and the high-order smoothness indicator $\tau_{z}$ is defined as follows:

(8) $$\tau_{z}=\left|{\beta_{3}}^{JS}-{\beta_{1}}^{JS}\right|=\left\{\begin{array}{c} O\left(h^{5}\right),\;{u^{\prime }}_{j}\neq 0\;\mathrm{or}\;{u^{\prime }}_{j}=0,\;{u^{\prime \prime }}_{j}\neq 0\\ O\left(h^{7}\right),\;{u^{\prime }}_{j}={u^{\prime \prime }}_{j}=0 \end{array}\right.$$

The redefined smoothness indicator for each stencil is represented as

(9) $${\beta_{k}}^{Z}=\frac{{\beta_{k}}^{JS}+\varepsilon }{{\beta_{k}}^{JS}+\tau_{z}+\varepsilon },\;k=1,2,3$$

The famous Z nonlinear weights from Jiang and Shu [25] are defined as

(10) $$\omega_{k}^{z}=\frac{\alpha_{k}^{z}}{\sum_{i=1}^{3}\alpha_{i}^{z}},\;\alpha_{k}^{z}=\frac{d_{k}}{{\beta_{k}}^{z}}=d_{k}\left(1+{\left(\frac{\tau_{z}}{{\beta_{k}}^{JS}+\varepsilon }\right)}^{q}\right),\;k=1,2,3$$

The order of the nonlinear weighting function at non-extreme points is

(11) $$\alpha_{k}^{z}=d_{k}+O\left({\left(h^{3}\right)}^{q}\right),\;k=1,2,3$$

This can naturally meet the requirement of fifth-order accuracy for q=1, and the order of the nonlinear weighting function at the extreme point is

(12) $$\alpha_{k}^{z}=\left\{\begin{array}{c} d_{k}+O\left({\left(h\right)}^{q}\right),\;u^{\prime }=0,\;{u^{\prime \prime }}_{j}\neq 0\\ d_{k}+O\left({\left(h^{2}\right)}^{q}\right),\;{u^{\prime }}_{j}={u^{\prime \prime }}_{j}=0 \end{array}\right.,\;k=1,2,3$$

when q≥2 can meet the accuracy requirements.

### 3.3. A New Fifth-Order Multi-Order Z Nonlinear Interpolation

Although Z weighting theoretically overcomes the problem of JS weighting decreasing at extreme points, in practical example testing, the nonlinear weight values of the sub-stencil often deviate from the linear weights on smooth regions, leading to smooth regions not achieving fifth-order accuracy. Inspired by the Z weighting high-order smoothing indicator, this article designs a nonlinear weighting method for the 5-3-3-3 stencils. The weighting method is completely consistent with JS, and the interpolation expression for the 5-point stencil adopts Equation (3). The smoothing indicators of each sub-stencil are

(13) $$\left\{\begin{array}{c} \beta_{0}^{MOZ}={\left({\beta_{1}}^{JS}-{\beta_{3}}^{JS}\right)}^{4}{\left({\beta_{1}}^{JS}+{\beta_{3}}^{JS}\right)}^{-3}\\ \beta_{1}^{MOZ}={\beta_{1}}^{JS}\\ \beta_{2}^{MOZ}={\beta_{2}}^{JS}\\ \beta_{3}^{MOZ}={\beta_{3}}^{JS} \end{array}\right.$$

The nonlinear weighting is called MOZ (multi-order-Z) candidate interpolation weighting since the order of the smoothness indicators for candidate interpolations of different orders is also different. Their orders of magnitude are

(14) $$\begin{array}{c} \beta_{0}^{MOZ}=\left\{\begin{array}{c} O\left(h^{14}\right),\;{u^{\prime }}_{j}\neq 0\;\\ O\left(h^{8}\right),\;{u^{\prime }}_{j}=0,{u^{\prime \prime }}_{j}\neq 0\\ O\left(h^{10}\right),\;u^{\prime }={u^{\prime \prime }}_{j}=0\; \end{array}\right.\\ \beta_{k}^{MOZ}=\left\{\begin{array}{c} O\left(h^{2}\right),\;{u^{\prime }}_{j}\neq 0\\ O\left(h^{4}\right),\;{u^{\prime }}_{j}=0,{u^{\prime \prime }}_{j}\neq 0\\ O\left(h^{6}\right),\;{u^{\prime }}_{j}={u^{\prime \prime }}_{j}=0 \end{array}\right.,\;k=1,2,3 \end{array}$$

The term $\beta_{0}^{MOZ}$ depends on all the nodes in the whole stencil. Near discontinuities,

${\left(|{\beta_{1}}^{JS}-{\beta_{3}}^{JS}|/\left({\beta_{1}}^{JS}+{\beta_{3}}^{JS}\right)\right)}^{3}=O\left(1\right)$. In smooth regions, ${\left(|{\beta_{1}}^{JS}-{\beta_{3}}^{JS}|/\left({\beta_{1}}^{JS}+{\beta_{3}}^{JS}\right)\right)}^{3}$ is a small quantity related to the grid scale. This design improves the order of the smoothness indicator of the 5-point sub-stencil, which increases the nonlinear weight of the 5-point sub-stencil in smooth regions to improve the scheme’s resolution and to avoid loss of extreme point accuracy.

The nonlinear weights of MOZ are specifically expressed as

(15) $$\left\{\begin{array}{c} \omega_{0}^{MOZ}=C_{T}\frac{{\left(\beta_{0}^{MOZ}\right)}^{p}}{{\left(\beta_{0}^{MOZ}+\varepsilon \right)}^{q}}\gamma_{0}\\ \omega_{k}^{MOZ}=C_{T}\frac{{\left(\beta_{0}^{MOZ}\right)}^{p}}{{\left(\beta_{k}^{MOZ}+\varepsilon \right)}^{q}}\gamma_{k} \end{array}\right.,k=1,2,3;p=q=1$$

where $\;C_{T}={\left[\gamma_{0}+\sum_{k=1}^{3}\gamma_{k}\frac{{\left(\beta_{0}^{MOZ}\right)}^{p}}{{\left(\beta_{k}^{MOZ}+\varepsilon \right)}^{q}}\right]}^{-1}$, and $\gamma_{0},\gamma_{k}$ are the linear weights, which satisfy $\gamma_{0}+\gamma_{1}+\gamma_{2}+\gamma_{3}=1$. Based on order analysis using Taylor series expansions, the leading terms of the nonlinear weights are

(16) $$\begin{array}{c} O\left(\omega_{0}^{MOZ}\right)=O\left(1\right)\\ O\left(\omega_{k}^{MOZ}\right)=O\left(\frac{\beta_{0}^{MOZ}}{\beta_{k}^{MOZ}+\varepsilon }\right)=\left\{\begin{array}{c} O\left(h^{10}\right),\;{u^{\prime }}_{j}\neq 0\;\\ O\left(h^{4}\right),\;{u^{\prime }}_{j}={u^{\prime \prime }}_{j}=0\;\\ O\left(h^{4}\right),\;{u^{\prime }}_{j}=0,{u^{\prime \prime }}_{j}\neq 0\; \end{array}\right.,\;k=1,2,3;\;p=q=1 \end{array}$$

The final nonlinear interpolation expression at the half node is

(17) $$u_{j+1/2}^{MOZ}=\sum_{k=0}^{3}\omega_{k}^{MOZ}u_{j+1/2}^{k}$$

in which $u_{j+1/2}^{k}$ is given by Equation (4). To ensure that the denominator is not zero, all subsequent examples in this article use $\varepsilon =10^{-20}$. Through accuracy analysis, it is easy to obtain that MOZ weights avoid degrading the accuracy at both first- and second-order extreme points. To improve the resolution near discontinuities, the linear weights of the three 3-point stencils are set to $\gamma_{k}=d_{k}\left(1-\gamma_{0}\right),k=1,2,3$, which can achieve fifth-order accuracy. In addition, it should be pointed out that based on the design concept of MOZ, it is easy to prove that if $\beta_{0}^{MOZ}=\tau_{z},p=2,q=2$ and the linear weights satisfy

(18) $$\left\{\begin{array}{c} \gamma_{0}=0.5,\\ \gamma_{k}=d_{k}\left(1-\gamma_{0}\right),\;k=1,2,3. \end{array}\right.$$

MOZ can degenerate into Z weighting; When the linear weights satisfy Equation (19) and p=1,q=2, it degenerates into JS weighting.

(19) $$\left\{\begin{array}{c} \gamma_{0}=0,\\ \gamma_{k}=d_{k}\left(1-\gamma_{0}\right),k=1,2,3. \end{array}\right.$$

To further illustrate the influence of the linear weight $\gamma_{0}$ on the nonlinear weight values of the 5-point sub-stencil in the MOZ weighting function, the variation of the nonlinear weight values at the first-order extreme points and discontinuities is given based on the following equation:

(20) $$u\left(x\right)=\left\{\begin{array}{c} e^{0.75(x-1)}x^{2},\;-1⩽x<0.525,\\ e^{0.75(x-1)}x^{2}+2,\;0.525⩽x⩽1. \end{array}\right.$$

**Figure 1:**
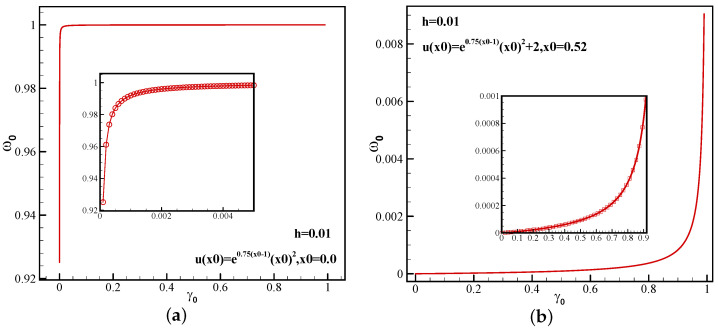
The variation of nonlinear weight $\omega_{0}^{MOZ}$ with linear weight $\gamma_{0}$ for 5-point stencil: (**a**) first-order extreme point; (**b**) discontinuities.

From Figure 1, it can be seen that although $\gamma_{0}$ is much smaller than the grid scale, the nonlinear weight $\omega_{0}^{MOZ}$ quickly approaches 1.0. In the vicinity of discontinuities, as $\gamma_{0}$ approaches 1.0, $\omega_{0}^{MOZ}$ is also much smaller than the grid scale. It can be seen that the MOZ designed in this paper achieves fifth-order accuracy and is relatively lenient with regard to the selection of linear weights.

### 3.4. Spectral and Accuracy Analysis

**Figure 2:**
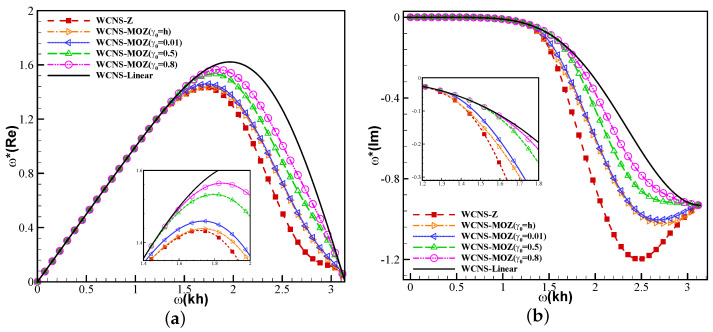
Results of spectral characteristics of nonlinear weights using MOZ and Z: (**a**) real part; (**b**) imaginary part. (Red square symbol denotes the result based on Z weights; Yellow right triangle, blue left triangle, green top triangle, and purple circle have MOZ weights of $\gamma_{0}=\left\{h,0.01,0.5,0.8\right\}$, respectively; Solid lines indicate linear format).

In this section, a simple analysis is conducted for comparing MOZ weighting with different linear weights and Z weighting in terms of accuracy and resolution. Using the ADR (approximate dispersion relation) [33] method, the spectral properties of fifth-order WCNS using ideal weights, Z weights, and MOZ weights with different linear weights are presented. Figure 2 shows the modified wavenumber between the ideal linear scheme and nonlinear weighted schemes. Figure 2a shows the real part, and Figure 2b shows the imaginary part. MOZ weighting with different linear weights has better dispersion and dissipation spectral characteristics than Z weighting.

The convergence accuracy of Z weighting and MOZ weighting with different linear weights are tested at extreme points using the function $u=e^{0.75(x-1)}x^{p}$[34]. The point x=0 is a first-order extreme point for p=2, while a second extreme point is p=3. Grid spacing of $h=2^{-m}h_{0}\left(h_{0}=0.04\right)$ is adopted. The accuracy test results are presented in Table 1 and Table 2. The MOZ weights successfully achieve the ideal order at both the first- and second-order extreme points. The Z function still degrades in order at the second-order extreme point. The actual errors of MOZ weighting are better than those of Z weighting, which are the same up to the third significant digits in Table 1 and Table 2.

In addition, the MOZ weights constructed in this article have the following two characteristics:

(1) Based on the original three 3-point stencils, a 5-point stencil is added and matched with a high-resolution high-order smoothness indicator. The choice of the smoothness indicator is consistent with the sub-stencil width. This choice helps the overall nonlinear interpolation to achieve the ideal order of accuracy. This choice ensures that despite the use of the JS weighting method, the format accuracy can still be maintained. In addition, by changing the linear weights, MOZ weighting can degrade to Z weighting and JS weighting, resulting in high design flexibility.
(2) The linear weights of three 3-point stencils are designed in an ideal allocation ratio of a convex combination to fifth-order accuracy, which increases the utilization of the sub-stencil information and improves the resolution near discontinuities.

## 4. Numerical Tests

The 4-stage fourth-order Runge–Kutta method in reference [35] is used for temporal discretization. To maintain the same temporal truncation error as the fifth-order spatial discretization, the time-marching step calculation method is

(21) $$\Delta t={\left(\frac{CFL}{a}h\right)}^{1.25},$$

where (a,CFL,h) are the maximum eigenvalue, the Courant number, and the minimum grid scale, respectively.

### 4.1. Linear Scalar Equation

(22) $$\frac{\partial u}{\partial t}+\frac{\partial u}{\partial x}=0.$$

Example 1: This example is calculated in $x\in \left[0,1\right]$ using periodic boundary conditions with initial conditions [30] of

(23) $$u\left(x,0\right)=e^{-300{\left(x-0.5\right)}^{2}}.$$

Based on the linear convection equation, the influence of MOZ weighting on the accuracy of the scheme is tested. Different from the accuracy tests, especially only for the extreme points in Section 3.4, the accuracy of the whole spatial and temporal discretization is tested here. The computational efficiency is compared, as shown in Table 3. The CPU time for MOZ is very close to that of Z.

**Figure 3:**
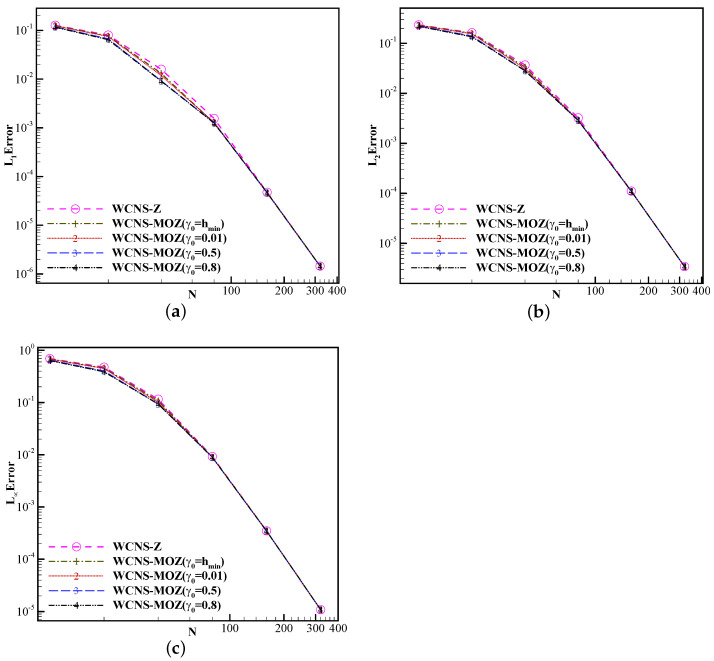
Convergence orders of fifth-order schemes based on Z and MOZ weighting with different linear weights at t=1 for the case of $u\left(x,0\right)=e^{-300{\left(x-0.5\right)}^{2}}$ based on the linear advection equation. (The horizontal axis represents the number of grid points (N) used in the calculation. The error of (**a**), (**b**), and (**c**) has already been provided in the previous text respectively. The circular symbol denotes the result of Z weighting; values of 1, 2, 3, and 4 denote the results of different linear weights of MOZ weighting).

Firstly, we test the impact of the linear weights $\gamma_{0}=\left\{h_{\min},0.01,0.5,0.8\right\}$ in MOZ weighting on the calculation results. Taking $u\left(x,0\right)=e^{-300{\left(x-0.5\right)}^{2}}$ as the initial value, the solution is advanced to the non-dimensional time of 1 using a grid spacing of $h=0.1\times 2^{-m}$ for the m-th level of the grid. Table 4 shows the errors and convergence orders of the results using MOZ weighting. $L_{1}$ is the average of the sum of errors of all grid points relative to the analytical solution. $L_{2}$ is the average of the sum of squares of errors of all grid points relative to the analytical solution. $L_{3}$ is the maximum value among the absolute values of errors of all grid points relative to the analytical solution. The different choices of the linear weights $\gamma_{0}$ do not affect the convergence order of the schemes. Figure 3 shows the error curves concerning grid resolutions using the three nonlinear weights. The resulting error of MOZ weighting with different linear weights is better than that of Z weighting.

**Figure 4:**
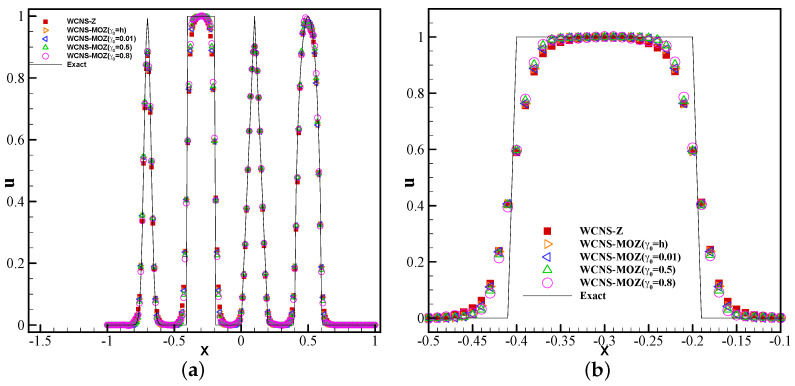
Test of three types of nonlinear weights based on piecewise discontinuous solutions using 200 grid points at t = 8: (**a**) variable u; (**b**) variable u zoomed in. (Red square symbol denotes the result based on Z weighting; yellow right triangle, blue left triangle, green top triangle, and purple circle have MOZ weightings of $\gamma_{0}=\left\{h,0.01,0.5,0.8\right\}$, respectively; solid lines denotes the exact solution).

Example 2: This example is calculated in $x\in \left[-1,1\right]$ using periodic boundary conditions with initial conditions [13]

(24) $$\begin{array}{c} u\left(x,0\right)=\left\{\begin{array}{c} \frac{1}{6}\left[G\left(x,z-\delta \right)-4G\left(x,z\right)+G\left(x,z+\delta \right)\right],x\in \left[-0.8,-0.6\right],\\ 1,x\in [-0.4,-0.2],\\ 1-\left|10x-1\right|,x\in \left[0,-0.2\right],\\ \frac{1}{6}\left[F\left(x,a-\delta \right)-4F\left(x,z\right)+F\left(x,a+\delta \right)\right],x\in \left[0.4,0.6\right],\\ 0,otherwise, \end{array}\right.\\ G\left(x,z\right)=e^{-\beta {\left(x-z\right)}^{2}},\\ F\left(x,a\right)=\sqrt{\max(1-\alpha^{2}{\left(x-a\right)}^{2},0)}, \end{array}$$

where $z=-0.7,\;\delta =0.005,\;\beta =\log2/(36\delta^{2}),\;a=0.5,\;\alpha =10$. The simulations are run to t=8. From Figure 4, it can be seen that Z weighting and MOZ weighting with different linear weights have almost no oscillations near discontinuities. Meanwhile, MOZ weighting exhibits higher resolution near extreme points and discontinuities than Z weighting.

### 4.2. Burgers’ Equation

The Burgers’ equation is a nonlinear scalar equation with the form

(25) $$\frac{\partial u}{\partial t}+\frac{\partial }{\partial x}\left(\frac{1}{2}u^{2}\right)=0.$$

This example is calculated in $x\in \left[0,2\right]$ using periodic boundary conditions with initial conditions [12]

(26) u(x,0)=sin(πx).

**Figure 5:**
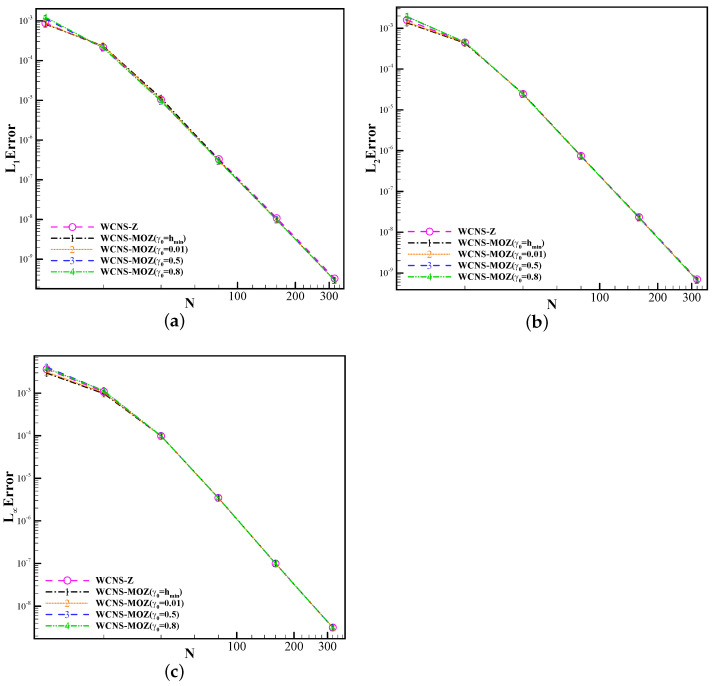
Convergence orders of four nonlinear fifth-order schemes at t=(0.5/π) of the one-dimensional inviscid Burgers’ equation with u(x,0)=sin(πx),t=(0.5/π) (The horizontal axis represents the number of grid points (N) used in the calculation. The error of (**a**), (**b**), and (**c**) has already been provided in the previous text respectively. The circular symbol denotes the result of Z weighting; values of 1, 2, 3, and 4 denote the results of different linear weights of MOZ weighting).

Table 5 and Figure 5 show the errors and convergence orders using four types of weights at t = 1 when discontinuities of the solution have not formed. From the test results, it can be seen that the convergence orders using Z and MOZ weights can converge to the fifth order. The errors of MOZ weights are similar to those of Z weights.

**Figure 6:**
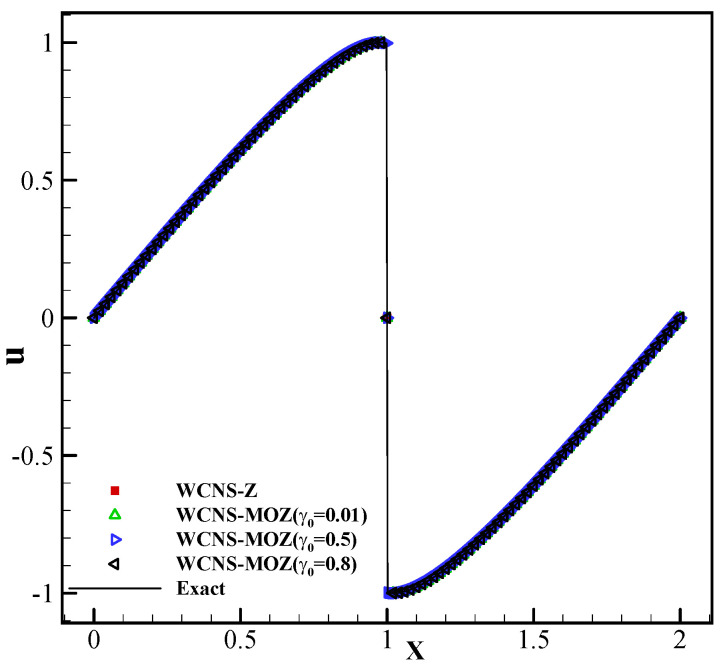
The results of four nonlinear fifth-order schemes of the one-dimensional inviscid Burgers’ equation with (x,0)=sin(πx) at t=1.5/π, grid N:100. (Red square symbol denotes the result based on Z weighting; yellow top triangle, blue right triangle, and green left triangle have MOZ weighting of $\gamma_{0}=\left\{0.01,0.5,0.8\right\}$, respectively; solid lines denote the exact solution.)

Discontinuity forms in the solution after t=1.5/π. From Figure 6, it can be seen that MOZ weights with different linear weights and Z weights effectively capture the discontinuity.

Next, the basic examples of one-dimensional and two-dimensional Euler equations are used to test the resolution and discontinuous capture ability of MOZ weighting and Z weighting. Based on the theoretical analysis and simple testing of scalar equations mentioned above, it is known that the linear weight of MOZ weighting will not affect the accuracy of the scheme, but the actual error will further decrease with the increase in linear weights $\gamma_{0}$. The following examples in this article were tested using linear coefficients of $\gamma_{0}=\left\{h,0.01,0.5,0.8\right\}$, and the test results of $\gamma_{0}$ with the smallest results are still better than those of Z weighting. The maximum $\gamma_{0}$ can also achieve stable discontinuous capture. Recommended values for linear weights are $h⩽\gamma_{0}⩽0.8$. By adjusting $\gamma_{0}$, the resolution and discontinuous capture ability of specific examples can be flexibly controlled and have a default value of $\gamma_{0}=0.5$. Due to limited space, we only provide test results for $\gamma_{0}=0.5$.

### 4.3. One-Dimensional Euler Equation

The Euler’ equation is a nonlinear scalar equation with the form

(27) $$\frac{\partial }{\partial t}\left(\begin{array}{c} \rho \\ \rho u\\ E \end{array}\right)+\frac{\partial }{\partial x}\left(\begin{array}{c} \rho u\\ \rho u^{2}+p\\ (E+p)u \end{array}\right)=0.$$

Example 1: The Sod and Lax problems [13] are both shock tube problems. The initial conditions of the Sod problem are

(28) $${\left(\rho ,u,p,\gamma \right)}^{T}=\left\{\begin{array}{c} {\left(1,0,1,1.4\right)}^{T},\;x\in \left[0,0.5\right),\\ {\left(0.125,0,0.1,1.4\right)}^{T},\;x\in \left[0.5,1.0\right], \end{array}\right.$$

while the initial conditions of the Lax problem are

(29) $${\left(\rho ,u,p,\gamma \right)}^{T}=\left\{\begin{array}{c} {\left(0.445,0.698,3.528,1.4\right)}^{T},\;x\in \left[-0.5,0\right),\\ {\left(0.5,0,0.571,1.4\right)}^{T},\;x\in \left[0,0.5\right]. \end{array}\right.$$

All the boundaries adopt the Dirichlet boundary condition using the left and right state values.

**Figure 7:**
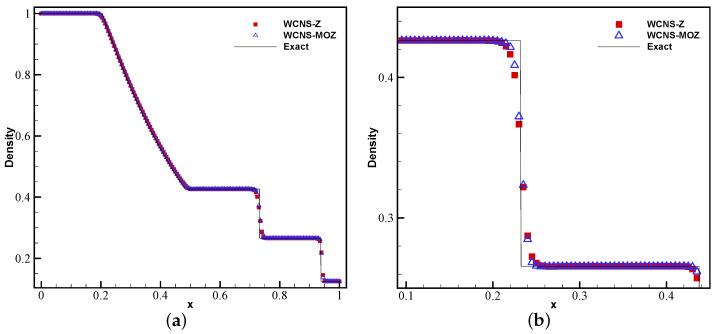
Numerical and exact solutions of the Sod problem at t = 0.25 using 200 grid points: (**a**) density; (**b**) density zoomed in (red square symbol denotes Z weighting, blue upper triangle denotes MOZ weighting, and solid line denotes the exact solution).

**Figure 8:**
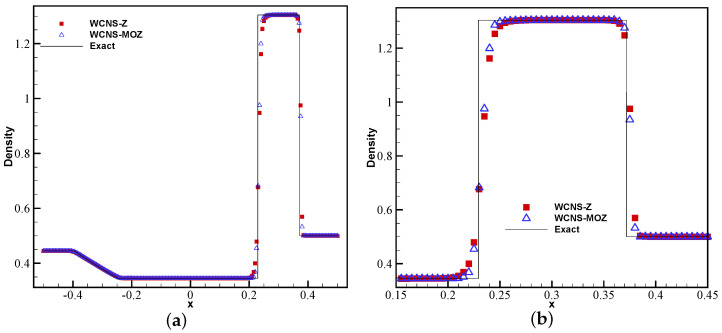
Numerical and exact solutions of the Lax problem at t = 0.15 using 200 grid points: (**a**) density; (**b**) density zoom in (red square symbol denotes Z weighting, blue upper triangle denotes MOZ weighting, and solid line denotes the exact solution).

Figure 7 and Figure 8 show the density results of nonlinear weighted functions at t = 0.25 on the Sod problem and t = 0.15 on the Lax problem, respectively. It can be seen that there are almost no non-physical oscillations near the discontinuity using Z weighting and MOZ weighting. The resolution of MOZ weighting near the discontinuity is higher than that of Z weighting.

Example 2: The Shu–Osher problem [25] has initial conditions of

(30) $${\left(\rho ,u,p,\gamma \right)}^{T}=\left\{\begin{array}{c} {\left(3.857,2.629,10.333,1.4\right)}^{T},\;x\in \left[0,1\right),\\ {\left(1+0.2\sin(5x),0,1,1.4\right)}^{T},\;x\in \left[1,10\right]. \end{array}\right.$$

All the boundaries adopt the Dirichlet boundary condition.

**Figure 9:**
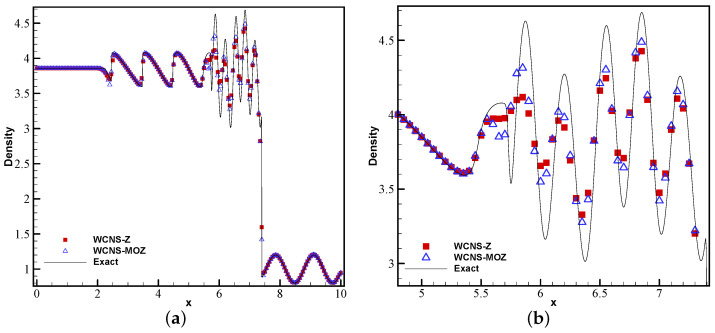
Numerical and exact solutions of the Shu–Osher problem at t = 1.8 using 200 grid points: (**a**) density; (**b**) density zoom in (red square symbol denotes Z weighting, blue upper triangle denotes MOZ weighting, and solid line denotes the exact solution).

Figure 9 shows the density results of nonlinear weighted functions at t = 1.8 on the Shu–Osher problem. From the enlarged density, it can be seen that MOZ weighting has better resolution for high-frequency waves compared to Z weighting, and it is closer to the exact solution.

### 4.4. Two-Dimensional Euler Equations

The equations have the form of

(31) $$\frac{\partial }{\partial t}\left(\begin{array}{c} \rho \\ \rho u\\ \rho v\\ E \end{array}\right)+\frac{\partial }{\partial x}\left(\begin{array}{c} \rho u\\ \rho u^{2}+p\\ \rho uv\\ (E+p)u \end{array}\right)+\frac{\partial }{\partial y}\left(\begin{array}{c} \rho v\\ \rho vu\\ \rho v^{2}+p\\ (E+p)v \end{array}\right)=0.$$

Example 1: Propagation of a vortex in two-dimensional space.A vortex is added to the mean flow ${\left(\rho ,u,v,p\right)}_{\infty }=\left(1,1,0,1\right)$, specified as

(32) $$\begin{array}{c} \left(\delta u,\delta v\right)=\varepsilon e^{\alpha \left(1-r^{2}\right)}\left(\bar{y},-\bar{x}\right),\\ \delta T=-\frac{\left(\gamma -1\right)\varepsilon^{2}}{4\gamma \alpha }e^{2\alpha \left(1-r^{2}\right)},\\ \delta S=0, \end{array}$$

where $\left(\bar{x},\bar{y}\right)=\frac{1}{Rc}\left(x-5,y-5\right),r^{2}={\bar{x}}^{2}+{\bar{y}}^{2},\varepsilon =0.3,Rc=0.5,\alpha =0.35$. The computations are performed on domain $\left[0,10\right]\times \left[0,10\right]$. The periodic boundary conditions are for all boundaries. The Courant number is 0.1, and the calculation is conducted until t = 1. Table 6 compare the accuracy of different weighting functions for solving this Euler problem at different grid resolutions. Although all weight functions can achieve the same convergence accuracy, the error of the MOZ weight is smaller than those of the other weight functions.

**Figure 10:**
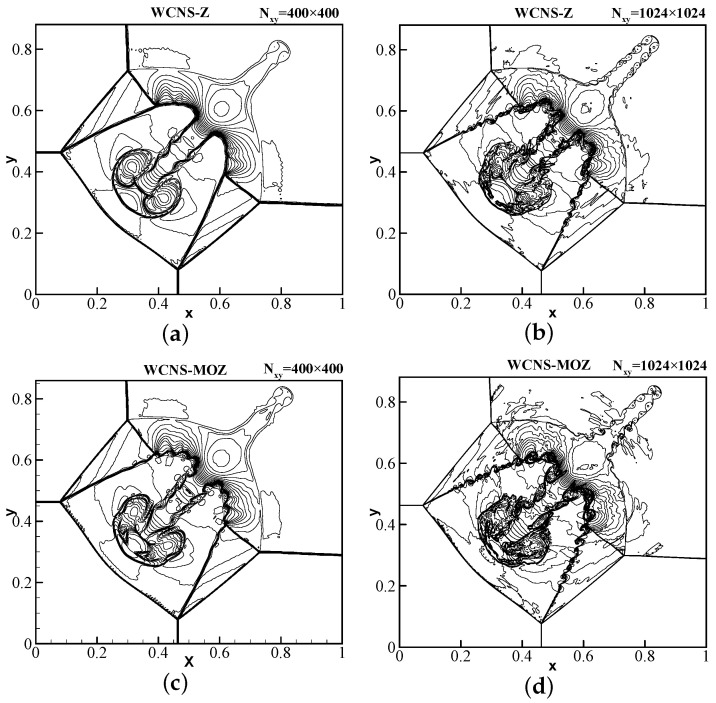
The density contour of 2D-Lax–Liu–Riemannian problem at t = 0.8 using 400 × 400 and 1024 × 1024 grid points. CFL = 0.3. The figures use 30 density contour lines ranging from 0.1–1.8. (**a**,**b**) Z; (**c**,**d**) MOZ.

The Courant number is 0.3, and calculation is conducted until t = 0.8. Figure 10 shows that the results using MOZ weighting have more vortices resolved at the shear layer compared to Z weighting at the same grid scale. With refinement of the grid, MOZ weighting can distinguish more small vortex structures compared to Z weighting in locations similar to the “mushroom-shaped” shape, resulting in a significant improvement in resolution. Example 2: A two-dimensional Riemannian problem [30] has initial conditions of

(33) $$\left(\rho ,u,v,p,\gamma \right)=\left\{\begin{array}{cc} \left(0.138,1.206,1.206,0.029,1.4\right) & \left(x,y\right)\in \left[0,\frac{4}{5}\right]\times \left[0,\frac{4}{5}\right],\\ \left(0.5323,0,1.206,0.3,1.4\right) & \left(x,y\right)\in \left[\frac{4}{5},1\right]\times \left[0,\frac{4}{5}\right],\\ \left(0.5323,1.206,0,0.3,1.4\right) & \left(x,y\right)\in \left[0,\frac{4}{5}\right]\times \left[\frac{4}{5},1\right],\\ \left(1.5,0,0,1.5,1.4\right) & \left(x,y\right)\in \left[\frac{4}{5},1\right]\times \left[\frac{4}{5},1\right], \end{array}\right.$$

Example 3: A double Mach reflection problem [35] has initial conditions

(34) $${\left(\rho ,u,p,\gamma \right)}^{T}=\left\{\begin{array}{c} {\left(1.4,0,0,1,1.4\right)}^{T},\;y<\sqrt{3}\left(x-1/6\right),\\ {\left(8.0,7.145,-4.125,116.5,1.4\right)}^{T},\mathrm{otherwise}. \end{array}\right.$$

**Figure 11:**
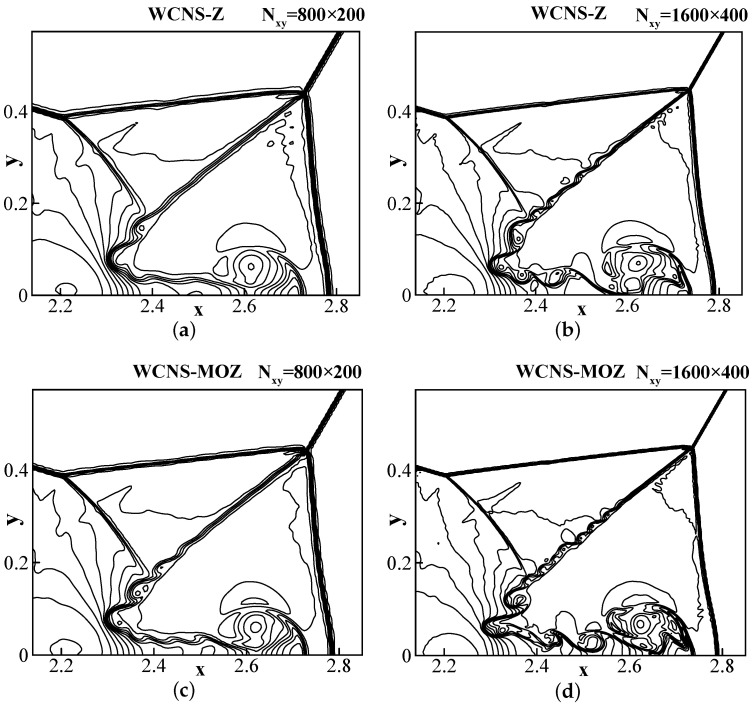
The density contour of double Mach reflection problem at t = 0.2 using 800 × 200 and 1600 × 400 grid points. CFL = 0.3. The figures use 30 density contour lines ranging from 1.5–22.7. (**a**,**b**) Z; (**c**,**d**) MOZ.

The initial conditions lead to a Mach 10 oblique shock with an angle of π/3 relative to the x-axis. The oblique shock is deflected by the leading edge of the solid wall at (x,y)=(1/6,0). The solid wall is extended to x=4 along the x-axis. The upper boundary is assigned by a Dirichlet boundary condition based on the analytical speed of the shock. The lower boundary of $x\in \left[0,1/6\right]$ uses an inviscid wall boundary. The left and right boundaries are, respectively, the inlet boundary conditions and the outlet boundary conditions. Using a Courant number of 0.3, the simulation is run until t = 0.8. From the enlarged contour of the shear layer regions in Figure 11, it can be seen that MOZ weighting shows better resolution compared to Z weighting. With refinement of the grid, this difference becomes more significant.

Example 4: A Rayleigh-Taylor instability problem [36] has initial conditions of

(35) $${\left(\rho ,u,v,p,\gamma \right)}^{T}=\left\{\begin{array}{c} (2,0,-0.025a\cos(8\pi x),1+2y,5/3),\;(x,y)\in [0,0.25]\times [0,0.5),\\ (1,0,-0.025a\cos(8\pi x),y+3/2,5/3),\;(x,y)\in [0,0.25]\times [0.5,1], \end{array}\right.$$

(36) $$\left(\rho ,u,v,p,\gamma \right)=\left\{\begin{array}{c} (1,0,0,2.5,5/3),\;y=1,\\ (2,0,0,1,5/3),\;y=0. \end{array}\right.$$

**Figure 12:**
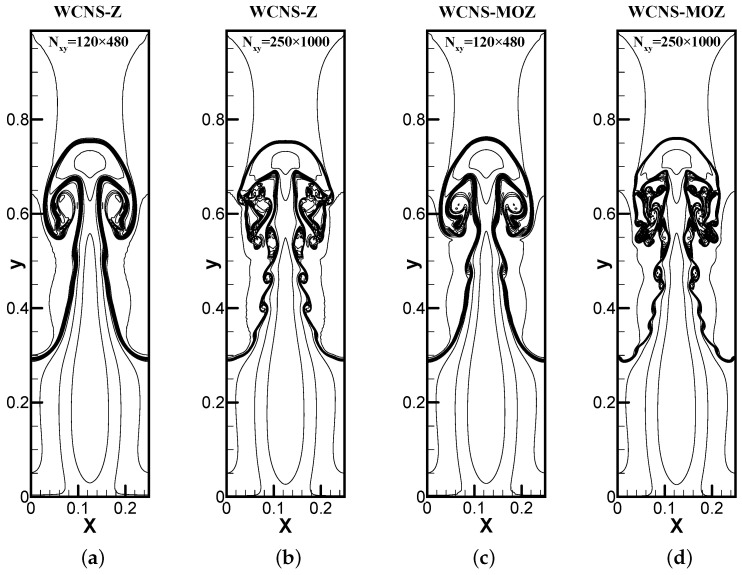
The density contour of Rayleigh–Taylor instability problem at t = 1.95 using 120 × 480 and 250 × 1000 grid points. CFL = 0.3. The figures use 30 density contour lines ranging from 0.9–2.2. (**a**,**b**) Z; (**c**,**d**) MOZ.

The upper and lower boundaries use Dirichlet boundary conditions according to Equation (36), while a symmetric boundary condition is adopted for the left and right boundaries. Using a Courant number of 0.3, the simulation is run until t = 1.95. Figure 12 shows that MOZ weighting has already captured the vortices near y = 0.5 on a 481 × 121 grid. However, these vortices have not yet fully formed when using Z weighting. With refinement of the grid, MOZ weighting can distinguish smaller-scale vortex structures near y = 0.6 compared to Z weighting. The results of MOZ weighting exhibit better resolution compared to Z weighting. Example 5: A Richtmyer–Meshkova instability problem [31] has initial conditions of

(37) $$\left(\rho ,u,v,p,\gamma \right)=\left\{\begin{array}{c} (3.33333,2.07063,0.0,7.125,1.4),\;0⩽x<0.06,\\ (1.0,0.0,0.0,1.0,1.4),\;0.06⩽x<0.1+0.008\cdot \cos(n\pi y),\\ (0.138,0.0,0.0,1.0,1.4),\;0.1\;+\;0.008\cdot \cos(n\pi y)⩽x⩽0.6. \end{array}\right.$$

**Figure 13:**
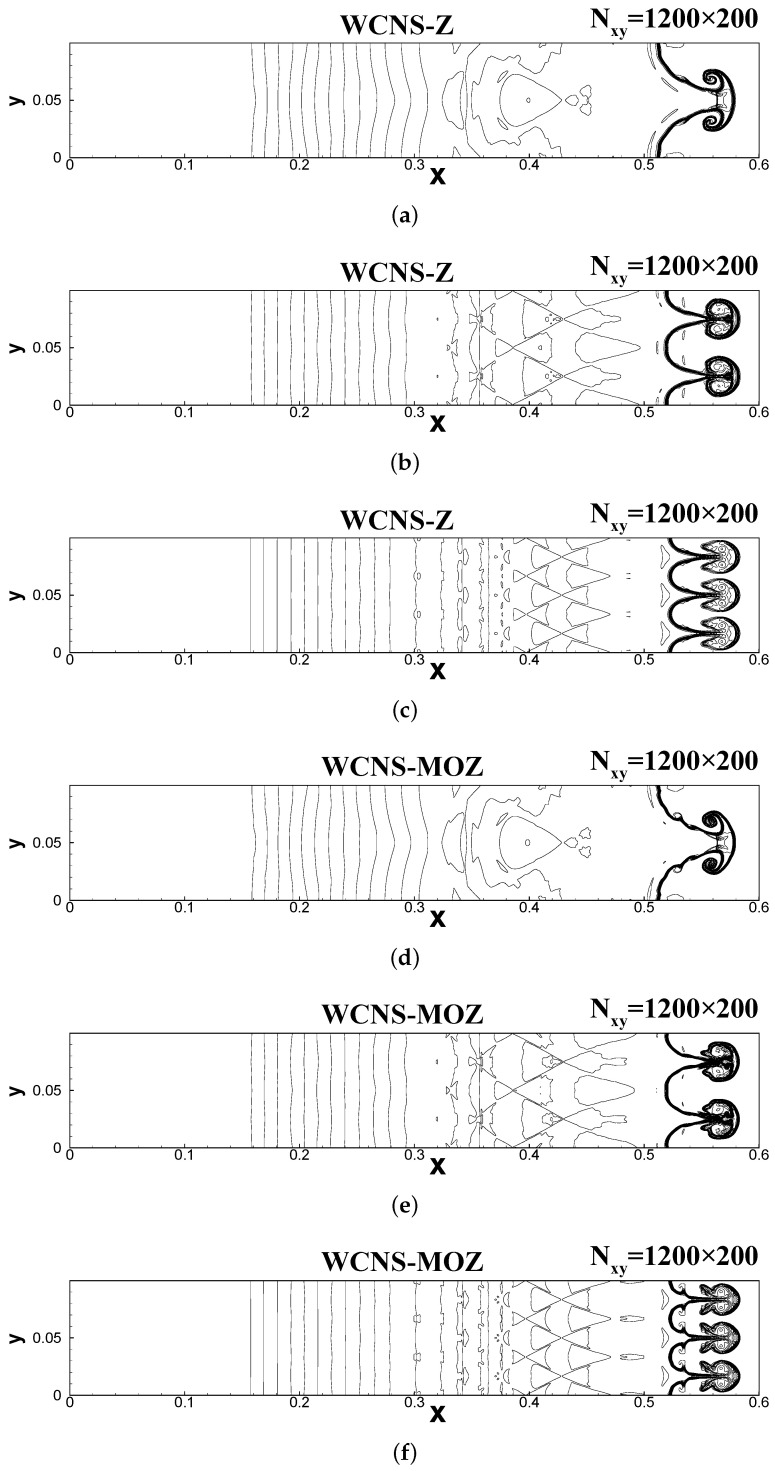
The density contour of the Richtmyer–Meshkova instability problem at t = 0.16 using 1200 × 200 grid points. CFL = 0.3. The figures use 30 density contour lines ranging from 0.2–3.2. (**a**,**c**,**e**) Results for Z weighting for cases with n = 20, 40, and 60, respectively; similarly, (**b**,**d**,**f**) are the results for MOZ weighting for cases with n = 20, 40, and 60, respectively.

The calculation domain is [0,0.6]×[0,0.1]. The upper and lower boundary conditions are periodic, and the left and right boundary conditions are fixed values that corresponds to the initial field. Using a Courant number of 0.3, the simulation is run until t = 0.16. Three scenarios are calculated: namely, 1, 2, and 3 shock bubbles, corresponding to n = 20, 40, and 60, respectively, in the initial conditions. From the results in Figure 13, it can be seen that the calculation results for all three cases show that MOZ weighting can capture more flow details about the “wake vortex” and “rod-shaped” positions of the shock bubble compared to Z-weighting, and its resolution is significantly better than that of Z weighting.

Example 6: Shock vortex interaction problem [37].

This example describes the interaction between a moving vortex and a steady shock wave. The calculation domain is $\left[0,2\right]\times \left[0,1\right]$. A shock wave with a Mach number of 1.1 is vertically located at x=0.5 and has values of $\left(\rho ,u,v,p\right)=\left(1,1.1\sqrt{\gamma },0,1\right)$ on the left-hand side, and the values on the right-hand side are calculated according to the shock relationship. The left and right boundaries are fixed values, and the top and bottom are symmetric boundaries. The vortex is initially located at x=0.5, and the vortex is described by

(38) $$\begin{array}{c} \tilde{u}=\varepsilon \tau_{v}e^{\alpha \left(1-{\tau_{v}}^{2}\right)}y,\\ \tilde{v}=-\varepsilon \tau_{v}e^{\alpha \left(1-{\tau_{v}}^{2}\right)}x,\\ \tilde{T}=-\frac{\left(\gamma -1\right)\varepsilon^{2}e^{2\alpha \left(1-{\tau_{v}}^{2}\right)}}{4\alpha \gamma },\\ \begin{array}{c} \tilde{S}=0,\\ \tau_{v}=\frac{r}{r_{c}}. \end{array} \end{array}$$

where $r=\sqrt{{\left(x-x_{c}\right)}^{2}+{\left(y-y_{c}\right)}^{2}}$ is the distance from the vortex center, $r_{c}=0.05$ is a parameter controlling the vortex size. ε=0.3 controls the strength of the vortex, and α=0.204 controls the attenuation rate of the vortices. The simulations are run until t = 0.8.

**Figure 14:**
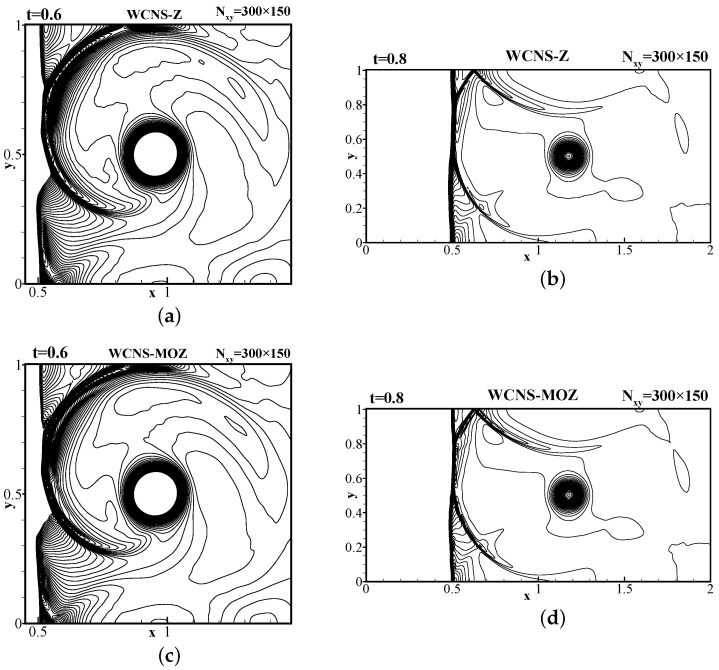
Pressure contour lines of shock vortex interaction problem using 300 × 150 grid points. (**a**,**b**) Present the pressure distribution of Z weighting at t = 0.6 and t = 0.8, respectively; (**c**,**d**) present the pressure distribution of MOZ weighting at t = 0.6 and t = 0.8, respectively. There are 60 pressure contour lines ranging from 1.19∼13.7. CFL = 0.3.

**Figure 15:**
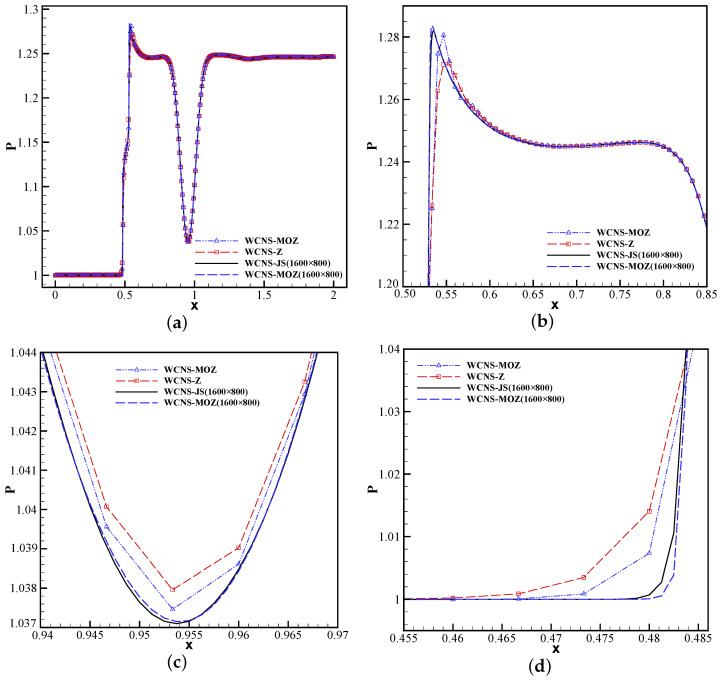
Pressure distribution for the line y = 0.5 across the center of the vortex at t = 0.6 using 300 × 150 grid points. (**a**) Pressure distribution using different weights. (**b**)Pressure zoomed in for the post-shock position. (**c**) Pressure zoomed in near the center of the vortex. (**d**) Pressure zoomed in for the pre-shock position. The reference solution is calculated using JS weights and 1600 × 800 grid points. (The red square symbol denotes Z weighting, the blue upper triangle denotes MOZ weighting, the blue long dash line denotes MOZ weighting (1600 × 800 grid points), the black solid line denotes the reference solution.)

Figure 14 shows that MOZ weighting can effectively simulate the shock. In addition, the results of Z weighting and MOZ weighting are compared at t = 0.6. Figure 15 shows the distribution of pressure at the vortex center y=0.5. Compared with the finer-grid result of JS weights, it can be seen that MOZ weights have lower dissipation, and no obvious overshoot occurs post the shock wave. MOZ weighting has higher resolution before and after shock waves than Z weighting.

## 5. Conclusions

Based on 5-3-3-3 stencil interpolation, a new high-order smoothness indicator for the fifth-order sub-stencil is proposed; the resulting multi-order weighting can maintain the ideal order of the (5-3-3-3) nonlinear weighting. The linear weight selection of three 3-point stencils satisfies the convex combination condition for the optimal accuracy of fifth-order interpolation. These strategies form multi-order-Z nonlinear weighting, which we denote as MOZ weighting. The main conclusions are as follows:

(1) Maintains accuracy: MOZ weighting ensures the ideal fifth-order accuracy of the scheme at the first- and second-order extremum points.
(2) High aspect ratio: Numerical validation tests show that the format using MOZ weighting has better resolution than Z weighting in smooth regions, shear layers, and discontinuities, which is consistent with the conclusions of spectral analysis, and there are no obvious non-physical false fluctuations at discontinuities. From a theoretical analysis perspective, the number of calculations for MOZ is equivalent to that required by Z weighting, so increasing resolution does not decrease computational efficiency, resulting in a significant improvement in the ratio of resolution to efficiency.

## Figures and Tables

**Table 1:** Errors and accuracy of nonlinear weighted interpolation method at first-order extreme point x=0 for the case $u=e^{0.75(x-1)}x^{2}$ for grids of $h=2^{-m}h_{0}\left(h_{0}=0.04\right)$.

	WCNS-Z		WCNS-MOZ $\gamma_{0}=h_{\min}$		WCNS-MOZ $\gamma_{0}=0.01$		WCNS-MOZ $\gamma_{0}=0.5$		WCNS-MOZ $\gamma_{0}=0.8$	
m	Error	Order	Error	Order	Error	Order	Error	Order	Error	Order
0	1.37 × 10^−6^		2.41 × 10^−8^		7.62 × 10^−9^		5.30 × 10^−9^		5.28 × 10^−9^	
1	2.92 × 10^−8^	5.55	1.66 × 10^−10^	7.18	1.66 × 10^−10^	5.52	1.66 × 10^−10^	4.99	1.66 × 10^−10^	4.99
2	6.71 × 10^−10^	5.44	5.23 × 10^−12^	4.99	5.23 × 10^−12^	4.99	5.23 × 10^−12^	4.99	5.23 × 10^−12^	4.99
3	1.71 × 10^−11^	5.30	1.64 × 10^−13^	5.00	1.64 × 10^−13^	5.00	1.64 × 10^−13^	5.00	1.64 × 10^−13^	5.00
4	4.73 × 10^−13^	5.18	5.12 × 10^−15^	5.00	5.12 × 10^−15^	5.00	5.12 × 10^−15^	5.00	5.12 × 10^−15^	5.00
5	1.38 × 10^−14^	5.10	1.60 × 10^−16^	5.00	1.60 × 10^−16^	5.00	1.60 × 10^−16^	5.00	1.60 × 10^−16^	5.00

**Table 2:** Errors and accuracy of nonlinear weighted interpolation method at second-order extreme point x=0 for the case $u=e^{0.75(x-1)}x^{3}$ for grids of $h=2^{-m}h_{0}\left(h_{0}=0.04\right)$.

	WCNS-Z		WCNS-MOZ $\gamma_{0}=h_{\min}$		WCNS-MOZ $\gamma_{0}=0.01$		WCNS-MOZ $\gamma_{0}=0.5$		WCNS-MOZ $\gamma_{0}=0.8$	
m	Error	Order	Error	Order	Error	Order	Error	Order	Error	Order
0	7.50 × 10^−4^		2.80 × 10^−8^		2.80 × 10^−8^		2.80 × 10^−8^		2.80 × 10^−8^	
1	1.84 × 10^−4^	2.03	8.86 × 10^−10^	4.98	8.86 × 10^−10^	4.98	8.86 × 10^−10^	4.98	8.86 × 10^−10^	4.98
2	4.35 × 10^−5^	2.08	2.78 × 10^−11^	4.99	2.78 × 10^−11^	4.99	2.78 × 10^−11^	4.99	2.78 × 10^−11^	4.99
3	9.44 × 10^−6^	2.20	8.73 × 10^−13^	5.00	8.73 × 10^−13^	5.00	8.73 × 10^−13^	5.00	8.73 × 10^−13^	5.00
4	1.94 × 10^−6^	2.28	2.73 × 10^−14^	5.00	2.73 × 10^−14^	5.00	2.73 × 10^−14^	5.00	2.73 × 10^−14^	5.00
5	4.30 × 10^−7^	2.18	8.55 × 10^−16^	5.00	8.55 × 10^−16^	5.00	8.55 × 10^−16^	5.00	8.55 × 10^−16^	5.00

**Table 3:** A comparison of CPU times obtained by solving the scalar equation with different nonlinear weights (the grid N is 1600).

Nonlinear Weights	JS	Z	MOZ
CPU time	0.484 s	0.516 s	0.531 s
Time ratio	1.000	1.066	1.097

**Table 4:** For $u\left(x,0\right)=e^{-300{\left(x-0.5\right)}^{2}}$, t=1, the error and order of Z weighting and different linear weights of MOZ weighting.

WCNS-MOZ$\left\{\gamma_{1}=h_{min}\right\}$						
Grid Point Number	$L_{1}$		$L_{2}$		$L_{\infty }$	
	Error	Order	Error	Order	Error	Order
11	1.25 × 10^−1^		2.31 × 10^−1^		6.83 × 10^−1^	
21	7.69 × 10^−2^	0.70	1.59 × 10^−1^	0.54	4.59 × 10^−1^	0.57
41	1.31 × 10^−2^	2.55	3.35 × 10^−2^	2.25	1.08 × 10^−1^	2.08
81	1.25 × 10^−3^	3.39	2.90 × 10^−3^	3.53	9.02 × 10^−3^	3.59
161	4.68 × 10^−5^	4.75	1.10 × 10^−4^	4.71	3.47 × 10^−4^	4.70
321	1.45 × 10^−6^	5.01	3.44 × 10^−6^	5.00	1.09 × 10^−5^	5.00
WCNS-MOZ $\left\{\gamma_{1}=0.01\right\}$						
Grid Point Number	$L_{1}$		$L_{2}$		$L_{\infty }$	
	Error	Order	Error	Order	Error	Order
11	1.24 × 10^−1^		2.30 × 10^−1^		6.79 × 10^−1^	
21	7.50 × 10^−2^	0.73	1.55 × 10^−1^	0.57	4.49 × 10^−1^	0.60
41	1.19 × 10^−2^	2.65	3.11 × 10^−2^	2.32	1.01 × 10^−1^	2.16
81	1.29 × 10^−3^	3.21	2.92 × 10^−3^	3.41	8.97 × 10^−3^	3.49
161	4.65 × 10^−5^	4.80	1.10 × 10^−4^	4.73	3.47 × 10^−4^	4.69
321	1.45 × 10^−6^	5.01	3.44 × 10^−6^	5.00	1.09 × 10^−5^	5.00
WCNS-MOZ$\left\{\gamma_{1}=0.5\right\}$						
Grid Point Number	$L_{1}$		$L_{2}$		$L_{\infty }$	
	Error	Order	Error	Order	Error	Order
11	1.18 × 10^−1^		2.22 × 10^−1^		6.48 × 10^−1^	
21	6.68 × 10^−2^	0.82	1.40 × 10^−1^	0.67	4.02 × 10^−1^	0.69
41	9.17 × 10^−3^	2.86	2.83 × 10^−2^	2.30	9.36 × 10^−2^	2.10
81	1.26 × 10^−3^	2.86	2.89 × 10^−3^	3.29	8.93 × 10^−3^	3.39
161	4.64 × 10^−5^	4.76	1.10 × 10^−4^	4.71	3.47 × 10^−4^	4.69
321	1.45 × 10^−6^	5.00	3.44 × 10^−6^	5.00	1.09 × 10^−5^	5.00
WCNS-MOZ$\left\{\gamma_{1}=0.8\right\}$						
Grid Point Number	$L_{1}$		$L_{2}$		$L_{\infty }$	
	Error	Order	Error	Order	Error	Order
11	1.16 × 10^−1^		2.19 × 10^−1^		6.36 × 10^−1^	
21	6.45 × 10^−2^	0.85	1.35 × 10^−1^	0.70	3.89 × 10^−1^	0.71
41	9.06 × 10^−3^	2.83	2.82 × 10^−2^	2.26	9.35 × 10^−2^	2.06
81	1.25 × 10^−3^	2.86	2.88 × 10^−3^	3.29	8.93 × 10^−3^	3.39
161	4.64 × 10^−5^	4.75	1.10 × 10^−4^	4.71	3.47 × 10^−4^	4.69
321	1.45 × 10^−6^	5.00	3.44 × 10^−6^	5.00	1.09 × 10^−5^	5.00
WCNS-Z						
Grid Point Number	$L_{1}$		$L_{2}$		$L_{\infty }$	
	Error	Order	Error	Order	Error	Order
11	1.27 × 10^−1^		2.34 × 10^−1^		6.95 × 10^−1^	
21	8.01 × 10^−2^	0.66	1.63 × 10^−1^	0.52	4.77 × 10^−1^	0.55
41	1.59 × 10^−2^	2.34	3.69 × 10^−2^	2.15	1.16 × 10^−1^	2.04
81	1.55 × 10^−3^	3.35	3.24 × 10^−3^	3.51	9.24 × 10^−3^	3.64
161	4.74 × 10^−5^	5.04	1.11 × 10^−4^	4.87	3.49 × 10^−4^	4.73
321	1.45 × 10^−6^	5.03	3.45 × 10^−6^	5.01	1.09 × 10^−5^	5.00

**Table 5:** For $u_{t}+{\left(\frac{u^{2}}{2}\right)}_{x}=0,u\left(x,0\right)=\sin\left(\pi x\right)$, t=0.5/π, the error and order of Z weighting and different linear weights of MOZ weighting.

WCNS-MOZ $\left\{\gamma_{1}=h_{min}\right\}$						
Grid Point Number	$L_{1}$		$L_{2}$		$L_{\infty }$	
	Error	Order	Error	Order	Error	Order
11	8.23 × 10^−4^		1.36 × 10^−3^		2.95 × 10^−3^	
21	2.33 × 10^−4^	1.82	4.34 × 10^−4^	1.65	9.74 × 10^−4^	1.60
41	1.14 × 10^−5^	4.36	2.50 × 10^−5^	4.12	9.88 × 10^−5^	3.30
81	3.11 × 10^−7^	5.19	7.37 × 10^−7^	5.08	3.47 × 10^−6^	4.83
161	9.79 × 10^−9^	4.99	2.27 × 10^−8^	5.02	9.96 × 10^−8^	5.12
321	2.95 × 10^−10^	5.05	6.60 × 10^−10^	5.11	3.15 × 10^−9^	4.98
WCNS-MOZ $\left\{\gamma_{1}=0.01\right\}$						
Grid Point Number	$L_{1}$		$L_{2}$		$L_{\infty }$	
	Error	Order	Error	Order	Error	Order
11	8.35 × 10^−4^		1.42 × 10^−3^		3.13 × 10^−3^	
21	2.33 × 10^−4^	1.84	4.48 × 10^−4^	1.66	1.02 × 10^−3^	1.61
41	1.03 × 10^−5^	4.50	2.45 × 10^−5^	4.19	9.88 × 10^−5^	3.37
81	2.97 × 10^−7^	5.12	7.32 × 10^−7^	5.06	3.47 × 10^−6^	4.83
161	9.79 × 10^−9^	4.93	2.27 × 10^−8^	5.01	9.96 × 10^−8^	5.12
321	2.95 × 10^−10^	5.05	6.60 × 10^−10^	5.11	3.15 × 10^−9^	4.98
WCNS-MOZ $\left\{\gamma_{1}=0.5\right\}$						
Grid Point Number	$L_{1}$		$L_{2}$		$L_{\infty }$	
	Error	Order	Error	Order	Error	Order
11	1.13 × 10^−3^		1.91 × 10^−3^		4.10 × 10^−3^	
21	2.08 × 10^−4^	2.44	4.58 × 10^−4^	2.06	1.14 × 10^−3^	1.85
41	9.54 × 10^−6^	4.45	2.92 × 10^−5^	4.24	9.88 × 10^−5^	3.52
81	2.96 × 10^−7^	5.01	7.31 × 10^−7^	5.05	3.47 × 10^−6^	4.83
161	9.79 × 10^−9^	4.92	2.27 × 10^−8^	5.01	9.96 × 10^−8^	5.12
321	2.95 × 10^−10^	5.05	6.60 × 10^−10^	5.11	3.15 × 10^−9^	4.98
WCNS-MOZ $\left\{\gamma_{1}=0.8\right\}$						
Grid Point Number	$L_{1}$		$L_{2}$		$L_{\infty }$	
	Error	Order	Error	Order	Error	Order
11	1.23 × 10^−3^		1.95 × 10^−3^		3.82 × 10^−3^	
21	2.09 × 10^−4^	2.56	4.56 × 10^−4^	2.10	1.14 × 10^−3^	1.74
41	9.54 × 10^−6^	4.45	2.42 × 10^−5^	4.23	9.88 × 10^−5^	3.53
81	2.96 × 10^−7^	5.01	7.31 × 10^−7^	5.05	3.47 × 10^−6^	4.83
161	9.79 × 10^−9^	4.92	2.27 × 10^−8^	5.01	9.96 × 10^−8^	5.12
321	2.95 × 10^−10^	5.05	6.60 × 10^−10^	5.11	3.15 × 10^−9^	4.98
WCNS-Z						
Grid Point Number	$L_{1}$		$L_{2}$		$L_{\infty }$	
	Error	Order	Error	Order	Error	Order
11	8.80 × 10^−4^		1.60 × 10^−3^		3.66 × 10^−3^	
21	2.18 × 10^−4^	2.02	4.49 × 10^−4^	1.83	1.08 × 10^−3^	1.76
41	1.04 × 10^−5^	4.39	2.46 × 10^−5^	4.19	9.89 × 10^−5^	3.45
81	3.31 × 10^−7^	4.97	7.46 × 10^−7^	5.04	3.47 × 10^−6^	4.83
161	1.08 × 10^−8^	4.93	2.35 × 10^−8^	4.98	9.96 × 10^−8^	5.12
321	3.29 × 10^−10^	5.04	7.01 × 10^−10^	5.07	3.15 × 10^−9^	4.98

**Table 6:** Errors and convergence orders based on JS, Z, and MOZ weights for two-dimensional Euler vortex simulation, at t=1.

WCNS-JS						
Grid Point Number	$L_{1}$		$L_{2}$		$L_{\infty }$	
	Error	Order	Order	Error	Order	Error
41	8.88 × 10^−5^		3.19 × 10^−4^		1.06 × 10^−5^	
81	1.16 × 10^−5^	2.94	8.05 × 10^−5^	1.99	2.94 × 10^−3^	0.44
161	1.33 × 10^−6^	3.13	1.06 × 10^−5^	2.92	2.78 × 10^−4^	3.40
321	8.41 × 10^−8^	3.98	6.84 × 10^−7^	3.95	1.88 × 10^−5^	3.89
641	2.46 × 10^−9^	5.09	2.13 × 10^−8^	5.00	8.64 × 10^−7^	4.44
WCNS-Z						
Grid Point Number	$L_{1}$		$L_{2}$		$L_{\infty }$	
	Error	Order	Order	Error	Order	Error
41	5.48 × 10^−5^		1.89 × 10^−4^		9.26 × 10^−6^	
81	9.01 × 10^−6^	2.61	6.74 × 10^−5^	1.49	2.34 × 10^−3^	0.31
161	1.11 × 10^−6^	3.02	9.26 × 10^−6^	2.86	2.39 × 10^−4^	3.29
321	5.60 × 10^−8^	4.31	5.47 × 10^−7^	4.08	1.88 × 10^−5^	3.67
641	9.79 × 10^−10^	5.84	1.08 × 10^−8^	5.66	4.95 × 10^−7^	5.25
WCNS-MOZ						
Grid Point Number	$L_{1}$		$L_{2}$		$L_{\infty }$	
	Error	Order	Order	Error	Order	Error
41	3.09 × 10^−5^		1.21 × 10^−4^		2.51 × 10^−6^	
81	3.52 × 10^−6^	3.13	2.37 × 10^−5^	2.36	5.77 × 10^−4^	1.62
161	2.93 × 10^−7^	3.58	2.51 × 10^−6^	3.24	5.60 × 10^−5^	3.37
321	7.17 × 10^−9^	5.35	7.27 × 10^−8^	5.11	2.61 × 10^−6^	4.43
641	7.81 × 10^−11^	6.52	4.23 × 10^−10^	7.43	1.21 × 10^−8^	7.75

## Data Availability

The data presented in this study are available on request from the corresponding author.

## Abbreviations

The following abbreviations are used in this manuscript:

MDPI	Multidisciplinary Digital Publishing Institute
DOAJ	Directory of open access journals
TLA	Three letter acronym
LD	Linear dichroism
